# The prevalence and determinants of use of vitamin D supplements among children in Alberta, Canada: a cross-sectional study

**DOI:** 10.1186/s12889-015-2404-z

**Published:** 2015-10-16

**Authors:** Lalani L. Munasinghe, Noreen Willows, Yan Yuan, Paul J. Veugelers

**Affiliations:** School of Public Health, University of Alberta, Population Health Intervention Research Unit, 3-50 University Terrace, 8303 112 Street, Edmonton, AB T6G2T4 Canada; Agricultural, Food & Nutritional Science, University of Alberta, 4-378 Edmonton Clinic Health Academy, 11405 87 Avenue, Edmonton, AB T6G1C9 Canada; School of Public Health, University of Alberta, 3-299 Edmonton Clinic Health Academy, 11405 87 Avenue, Edmonton, AB T6G1C9 Canada

**Keywords:** Vitamin D, Children, Supplements, Multivitamins, Public health, Determinants

## Abstract

**Background:**

Limited cutaneous synthesis due to low sun exposure and inadequate dietary intake makes vitamin D supplementation a necessity for many Canadian children. Identification of the factors associated with supplement use is necessary for public health awareness campaigns, but they have not been identified previously. Therefore, the purpose of this study was to assess the prevalence and the determinants of the use of vitamin D supplements among children in the province of Alberta, Canada.

**Methods:**

In 2014, a representative sample of grade five students (10–11 y) in Alberta (*n* = 2686) was surveyed. Data on dietary intake and use of vitamin D supplements were obtained using a modified Harvard Youth/Adolescent Food Frequency questionnaire. Mixed effect multiple logistic regression was employed to identify the key correlates of supplement use.

**Results:**

Use of vitamin D supplements by children was 29.45 % although only 11.83 % took supplements daily. Children who resided in a metropolitan area (OR = 1.32; 95 % CI:1.06–1.65), were more physically active (2nd tertile: OR = 1.39; 95 % CI:1.09–1.78 and 3rd tertile: OR = 1.70; 95 % CI:1.33–2.16), or whose parents completed college (OR = 1.35; 95 % CI:1.05–1.74) were more likely to take vitamin D supplements. Prevalence of use was highest among those who had a high vitamin D diet and those with under/normal body weight status, although supplement use was not statistically associated with either dietary vitamin D intake or weight status.

**Conclusions:**

A considerable proportion of children did not take vitamin D supplements. Region of residence, physical activity level and parental education were determinants of supplement use, independent of child’s gender, household income, weight status and dietary practices. We suggest prioritizing public health efforts to support strategies to make parents aware of the importance of providing the correct dose of vitamin D supplements for their children to meet dietary recommendations.

## Background

The 2012/2013 Canadian Health Measure Survey [1] revealed that 35 % of all Canadians and more than 20 % of children are at risk of poor bone health. This is presumably due to limited cutaneous synthesis of vitamin D through sun exposure because of Canada’s high latitude [2, 3] and poor dietary intake of vitamin D rich foods [3–6]. Obese and overweight children are potentially more susceptible for poor vitamin D status [1, 7] because of sequestration of vitamin D into a larger pool of adipose tissues in the body [8, 9]. Efficacy of the mandatory vitamin D fortification of designated staple foods in Canada [10, 11] to ensure vitamin D adequacy is low due to under-fortification [12] and insufficient consumption [4, 12]. Therefore, meeting current dietary guidelines for vitamin D that includes an estimated average requirement of 400 IU/day and a recommended dietary allowance of 600 IU/day for children over 1 year of age [13] is difficult without supplementation. Canadians who use supplements are more likely to maintain adequate vitamin D levels [7, 14]. Studies [2, 6, 14–16] have emphasized the importance of supplementation as a strategy to overcome the issue of poor vitamin D status. Although all Canadians might benefit from supplements to maintain vitamin D levels [14], Health Canada [17] only recommends a daily vitamin D supplement of 400 IU for breastfed, healthy term infants and adults over the age of 50. There are no recommendations for the vitamin D supplementation of children.

Studies have identified age [18, 19], gender [20], weight status [21], socio-economic status [19, 21], level of physical activity [20–23], quality of diet [21, 22], and parental use of supplements [18] to be associated with the use of multivitamin/mineral supplements among children. Although the factors associated with multivitamin/mineral supplement use are well studied, those associated with vitamin D supplement use among children have not been given attention. In Canada, vitamin D supplements for children are available as vitamin D supplements (containing only vitamin D) and vitamin D-containing multivitamins. However, it is advisable to use vitamin D supplements as cholecalciferol to meet dietary guidelines due to the varying forms of vitamin D (i.e., ergocalciferol and cholecalciferol) and amount of vitamin D in multivitamins. Given that the determinants of use can vary according to the type of supplement being considered [24], the factors associated with the use of vitamin D supplements need to be studied. Awareness of such factors is crucial to inform policy decision-makers and stakeholders in planning programs to promote the optimal vitamin D status of the population through supplementation. The purpose of this study was to determine the prevalence of vitamin D supplement use and the associated factors among children in Alberta. Unlike use of vitamin D supplements, the use of multivitamins is quite common among developed nations [19, 23, 24]. Therefore, we also aimed to identify if there were differences in the factors associated with vitamin D supplement use with those of multivitamin/mineral use. This information would be valuable in establishing whether programs for promoting vitamin D use would need to be different from programs promoting supplement use in general.

## Methods

### The survey and subjects

We analyzed data on demographic and socio-economic factors, diet and supplements, and physical activity that were collected as part of the “Raising healthy Eating and Active Living Kids in Alberta” (REAL Kids Alberta) survey, a population-based study of grade five students (age 10–11 years) and their parents in the province of Alberta, Canada in 2014. A one-stage stratified random sample of 140 elementary schools across Alberta was selected. The sampling frame included all elementary schools in Alberta with grade five students with the exception of private schools, francophone schools, on-reserve federal schools, charter schools and colony schools. Therefore, 90.2 % of all elementary students in Alberta were eligible for random selection based on three geographical strata (metropolitan, urban, and rural). Schools were randomly selected within each stratum to achieve proportional representation [25]. More details on the project aim and the measures used are available on the project’s website: http://www.realkidsalberta.ca. A total of 4993 home booklets and parental consent forms were sent home with students to be completed by parent(s) or guardian(s) and returned to school. Among 3284 booklets returned, 2958 students were granted consent and participated in the study (participation rate = 59 %). Students who did not complete the food frequency questionnaires (*n* = 107) and 46 with missing data on use of multivitamins and/or vitamin D supplements were excluded from the present analysis. Students who had reported energy intakes of <500 kcal and >5000 kcal were excluded (*n* = 119) as per established criteria when food frequency questionnaire data is involved [26]. Therefore, the analysis of the present study was restricted to a total of 2686 students (53.8 %).

### Outcome of interest: vitamin D-containing supplement use

Information on use of vitamin D supplements and multivitamins was obtained from a modified Harvard Youth/Adolescent Food Frequency Questionnaire (YAQ). Under the guidance of a trained evaluation assistant, each student completed the YAQ during classroom time in a school day. In separate questions, students were asked, “Do you now take vitamin D supplements (pills/drops)?”, “Do you now take multivitamins?”, “How often did you take vitamin D supplements over the past year?” and “How often did you take multivitamins over the past year?”. “Vitamin D supplement users” were defined as a child who reported ever taking a vitamin D supplement and “multivitamin supplement users” a child who reported ever taking a multivitamin.

### Anthropometric, demographic, socio-economic, and lifestyle determinants

Evaluation assistants measured weight and standing height from all children to calculate Body Mass Index (BMI) as weight divided by height^2^ (kg/m^2^). Weight was measured to the nearest 0.1 kg using calibrated digital scales (Health-o-meter, USA) and height was measured to the nearest 0.1 cm using stadiometers (Seca-Stadiometers, Germany). Overweight and obesity were defined according to the age- and gender- specific cut-offs of the International Obesity Task Force BMI for children and youth [27]. Region of residence was defined based on the schools located in three geographical locations: metropolitan (Calgary and Edmonton, cities with a population of about 1 million people each), urban (other municipalities with more than 40,000 residents) and rural (municipalities with less than 40,000 residents). Data on the level of parent education attainment and household income were collected from parent responses in the home survey. Physical activity level (PAL) was identified as a single physical activity score ranging from 0 to 5 that was derived from a 29-item questionnaire adapted from the Physical Activity Questionnaire for Older Children (PAQ-C), which has been validated for children [28]. The physical activity questionnaire contains questions about active transportation, activity level inside and outside of school hours, participation in sports and other forms of active play. Diet quality was derived using the Diet Quality Index International, a composite measure that encompasses dietary adequacy, variety, moderation, and balance ranging from 0 to 100 [29]. Dietary intake data were obtained based on responses to the questions in validated YAQ [30] that assesses diets of those 9–18 years of age. Total dietary vitamin D intake and total calorie intake from food were calculated using Canadian Nutrient File [31].

### Statistical analyses

Data were analyzed using Stata version 13 (Stata Corp, College Station, TX, USA). Descriptive statistics were used to characterize the population and to identify the frequencies of children using vitamin D supplements and multivitamins. Mixed effect multiple logistic regression models with children nested in schools identified the association of weight status, lifestyle, socioeconomic, and demographic factors with use of vitamin D supplements and multivitamins. Akaike Information Criteria and Bayesian Information Criteria were employed to select the parsimonious multiple regression models. Missing data were treated as a separate category. Data analyses were adjusted for total energy intake when food frequency data were involved as per established criteria [26]. All analyses were weighted to represent unbiased provincial estimates of the grade five student population in Alberta. The Health Research Ethics Board at the University of Alberta approved this study, including data collection and parental informed consent forms.

## Results

Altogether, 62.14 % of grade five students (age 10–11 years) in Alberta participating in this study took only vitamin D supplement (8.06 %), only multivitamin (32.69 %), or both a vitamin D supplement and multivitamin (21.39 %). The characteristics of students taking vitamin D supplements (29.45 %) and multivitamins (54.08 %) are presented in Table 1. However, of grade five students, only 11.83 % took vitamin D supplements and 28.43 % took multivitamins on a daily basis during the past year.

**Table 1:** General characteristics and the prevalence of supplement use of 10–11 year-old students in Alberta, Canada

	All students, %^a^ (*n* = 2686)	Vitamin D supplement users (with or without multivitamin use), % (*n* = 769)^a^	Multivitamin supplement users (with or without vitamin D supplement use), % (*n* = 1468)
Gender			
Girls	53.49	51.81	54.57
Boys	46.51	48.19	45.43
Parental education^b^			
Secondary or less	23.19	19.68	21.07
College	33.54	35.87	34.28
University/graduate	37.89	38.90	39.99
Household income			
≤$50,000	13.21	12.82	11.55
$50,001 – $100,000	19.03	19.64	20.09
≥$100,001	28.89	30.07	30.81
Non-disclosed/Missing^c^	38.87	37.48	37.55
Region of residence			
Rural	39.55	35.87	40.19
Urban	8.28	8.14	8.65
Metropolitan	52.17	55.99	51.16
Weight status^b^			
Under/normal weight	68.59	71.30	70.55
Overweight	20.84	18.38	19.61
Obese	7.86	7.58	6.76
Physical activity level			
1st Tertile	33.32	27.85	30.94
2nd Tertile	33.32	34.11	32.97
3rd Tertile	33.36	38.04	36.09
Energy-adjusted diet quality index^d^			
1st Tertile	33.32	30.76	31.09
2nd Tertile	33.32	34.43	34.37
3rd Tertile	33.36	34.81	34.54
Energy-adjusted total dietary vitamin D^d^			
1st Tertile	33.32	33.73	32.77
2nd Tertile	33.32	31.76	31.64
3rd Tertile	33.36	34.51	35.59

^a^Results were weighted to represent provincial estimates of the grade five student population (age: 10–11y) in Alberta

^b^<5 % of missing data

^c^26.63 % non-disclosed responses (participants were provided option not to disclose their household income) and 12.23 % missing data

^d^“Energy adjusted” DQI and dietary vitamin D intake were computed as the residuals from the regression model with total energy intake as the independent variable and absolute DQI or dietary vitamin D intake as the dependent variable as per established criteria [26]

Table 2 depicts the associations of demographic, socio-economic, anthropometric and life style factors on the use of vitamin D supplements. Students whose parents completed education up to college level were more likely to take vitamin D supplements as compared to those whose parents completed secondary school education or less (univariable: OR = 1.38; 95 % CI = 1.06, 1.78 and parsimonious: OR = 1.35; 95 % CI = 1.05, 1.74). Students residing in a metropolitan area were more likely to take vitamin D supplement as compared to those attending schools in rural areas (univariable: OR = 1.27; 95 % CI = 1.03, 1.56 and parsimonious: OR = 1.32; 95 % CI = 1.06, 1.65). PAL was highly correlated with vitamin D supplement use in both univariable (1st Tertile: OR = 1.39; 95 % CI = 1.09, 1.77 and 2nd Tertile: OR = 1.68; 95 % CI = 1.33, 2.14) and parsimonius (1st Tertile: OR = 1.39; 95 % CI = 1.09, 1.78 and 2nd Tertile: OR = 1.70; 95 % CI = 1.33, 2.16) models. Table 3 shows that parental education, household income and PAL were associated with multivitamin use. Energy-adjusted dietary vitamin D intake was not retained in the parsimonious models for either type of supplement (vitamin D supplement in Table 2 or multivitamin in Table 3).

**Table 2:** Determinants of vitamin D supplement use among 10–11-year-old students in Alberta, Canada^a^

	Univariable model	Parsimonious model^b^
	Odds ratio (95 % CI)	Odds ratio (95 % CI)
Demographic, socio-economic and anthropometric factors		
Gender		
Girls	1.00	1.00
Boys	1.10 (0.91, 1.33)	1.03 (0.85, 1.25)
Parental education		
Secondary or less	1.00	1.00
College	1.38 (1.06, 1.78)*	1.35 (1.05, 1.74)*
University or graduate	1.29 (1.00, 1.65)*	1.21 (0.94, 1.56)
Household income^c^		
≤$50,000	1.00	1.00
$50,001–100,000	1.08 (0.80, 1.47)	1.08 (0.79, 1.48)
≥$100,001	1.08 (0.80, 1.48)	1.03 (0.74, 1.43)
Region of residence		
Rural	1.00	1.00
Urban	1.11 (0.91, 1.37)	1.13 (0.92 1.40)
Metropolitan	1.27 (1.03, 1.56)*	1.32 (1.06, 1.65)*
Weight status		
Under/normal weight	1.00	1.00
Overweight	0.80 (0.64, 1.00)*	0.82 (0.66, 1.04)
Obese	0.91 (0.60, 1.40)	0.93 (0.60, 1.43)
Lifestyle factors		
Physical activity level		
1st Tertile	1.00	1.00
2nd Tertile	1.39 (1.09, 1.77)**	1.39 (1.09, 1.78)**
3rd Tertile	1.68 (1.33, 2.14)***	1.70 (1.33, 2.16)***
Energy-adjusted diet quality index^d^		
1st Tertile	1.00	1.00
2nd Tertile	1.16 (0.91, 1.47)	1.12 (0.89, 1.42)
3rd Tertile	1.18 (0.95, 1.46)	1.10 (0.89, 1.36)
Energy-adjusted dietary vitamin D intake^d^		
1st Tertile	1.00	–
2nd Tertile	0.93 (0.73, 1.19)	
3rd Tertile	1.03 (0.81, 1.31)	

^a^Results were weighted to represent provincial estimates of the grade five student population (age: 10-11y) in Alberta. Vitamin D supplement users were defined as those who used vitamin D supplements irrespective of use of multivitamins

^b^Adjusted for demographic, socio-economic and anthropometric factors in the table

^c^26.63 % non-disclosed responses (participants were provided option not to disclose their household income) and 12.23 % missing data

^d^“Energy adjusted” DQI and dietary vitamin D intake were computed as the residuals from the regression model with total energy intake as the independent variable and absolute DQI or dietary vitamin D intake as the dependent variable as per established criteria [26]

*p <0.05

**p <0.01

***p <0.001

**Table 3:** Determinants of use of multivitamins among of 10–11-year-old students in Alberta, Canada^a^

	Univariable model	Parsimonious model^b^
	Odds ratio (95 % CI)	Odds ratio (95 % CI)
Demographic, socio-economic and anthropometric factors		
Gender		
Girls	1.00	1.00
Boys	0.91 (0.75, 1.10)	0.85 (0.70, 1.03)
Parental education		
Secondary or less	1.00	1.00
College	1.26 (1.02, 1.55)*	1.25 (1.02, 1.54)*
University or graduate	1.34 (1.07, 1.69)*	1.33 (1.06, 1.68)*
Household income^c^		
≤$50,000	1.00	1.00
$50,001–100,000	1.48 (1.10, 2.00)**	1.43 (1.05, 1.94)*
≥$100,001	1.44 (1.09, 1.91)**	1.29 (0.97, 1.71)
Region of residence		
Rural	1.00	1.00
Urban	1.06 (0.81, 1.40)	1.06 (0.81 1.39)
Metropolitan	0.92 (0.73, 1.17)	0.95 (0.76, 1.19)
Weight status		
Under/normal weight	1.00	1.00
Overweight	0.84 (0.67, 1.05)	0.88 (0.70, 1.09)
Obese	0.70 (0.50, 0.98)*	0.76 (0.54, 1.08)
Lifestyle factors		
Physical activity level		
1st Tertile	1.00	1.00
2nd Tertile	1.21 (0.99, 1.47)	1.20 (0.97, 1.47)
3rd Tertile	1.55 (1.26, 1.90)***	1.56 (1.27, 1.91)***
Energy-adjusted diet quality index^d^		
1st Tertile	1.00	1.00
2nd Tertile	1.22 (0.99, 1.51)	1.20 (0.97, 1.49)
3rd Tertile	1.22 (0.97, 1.55)	1.13 (0.90, 1.43)
Energy-adjusted dietary vitamin D intake^d^		
1st Tertile	1.00	–
2nd Tertile	0.95 (0.77, 1.17)	
3rd Tertile	1.16 (0.97, 1.39)	

^a^Results were weighted to represent provincial estimates of the grade five student population (age: 10-11y) in Alberta. Multivitamin users were defined as those who used multivitamins irrespective of use of vitamin D supplements

^b^Adjusted for demographic, socio-economic and anthropometric factors in the table

^c^26.63 % non-disclosed responses (participants were provided option not to disclose their household income) and 12.23 % missing data

^d^“Energy adjusted” DQI and dietary vitamin D intake were computed as the residuals from the regression model with total energy intake as the independent variable and absolute DQI or dietary vitamin D intake as the dependent variable as per established criteria [26]

*p <0.05

**p <0.01

***p <0.001

## Discussion

This study indicated that approximately one third of school children aged 10–11 years in Alberta took vitamin D supplements and approximately half of them took multivitamins. Parental education, region of residence and PAL were determinants of vitamin D supplement use among children, independent of child’s gender, household income, weight status and dietary practices. Both vitamin D and multivitamin supplement use were more prevalent among physically active children and those from families with high socioeconomic status, and use was less common among boys than among girls.

Consistent with other findings [19, 23, 24], we identified that children in Alberta were more likely to use multivitamins than vitamin D supplements. Although the use of supplements (vitamin D supplements, multivitamins, or both) was twice as common among children in our study (62 %) compared to the national averages in 2007/2009 of 31 % [32] and in 2009/2011 of 34 % [7], only small proportions of children were taking supplements once a day, or more frequently. Therefore, promotion of supplements on a daily basis is essential to meet the recommended daily requirement of vitamin D.

Parental education was associated with use of both vitamin D supplements and multivitamins. We did not find any association of vitamin D supplement use with household income, whereas multivitamin use in our study and other studies [11, 19, 21] had a positive association with income. Therefore, unlike multivitamin use, vitamin D supplement use mainly depended on the knowledge of the parents, independent of their income. Students residing in a metropolitan area were more likely to take vitamin D supplements than those attending schools in rural area. However, residential area was not associated with multivitamin supplement use, and most of the multivitamins available for children in Canada contain only half of the recommended daily amount of vitamin D. Parents may have been unaware of the vitamin D composition in multivitamins or the importance of vitamin D for their children. Therefore, dissemination of public health knowledge on children’s need for sole vitamin D supplements based on residential area appears to be important.

Vitamin D status of Canadians mainly depends on the diet and supplements [33] due to limited cutaneous synthesis [2, 3]. Among children in Alberta, the prevalence of both vitamin D supplement and multivitamin use were low among those who consumed less vitamin D from the diet (30–35 % in each tertile); therefore approximately 65–70 % of the children with a low intake of dietary vitamin D were at risk of deficiency. However, we did not find any significant association in the regression analysis of dietary vitamin D intake and quality of the diet with supplement use. Some other findings on multivitamin supplement use [21, 22] demonstrated that children with better quality diets were more likely to take either vitamin D or multivitamin supplements. We previously identified that only one-fifth of the students in our study met dietary guidelines for vitamin D through both diet and supplements [34]. Although it is important to encourage children to adopt healthy eating behaviours in addition to taking supplements for adequate nutrient intake [22], our findings reveal the importance of using vitamin D supplements independent of diet quality. Public health strategies aimed at improving the use of vitamin D supplements need to make parents also aware of the importance of consuming vitamin D rich dietary sources.

Valtueña et al. [35] identified the interactions between vitamin D and PAL in two possible directions, i.e., sufficient vitamin D levels improve bone health only in active children or PAL improves bone health in individuals with sufficient vitamin D levels. Therefore, predominant use of vitamin D supplements by more active children in our study is notably interesting as proper vitamin D levels potentially better benefit the active children. Obese and overweight children may need extra vitamin D [36] to compensate for their additional requirements. They are susceptible to poor vitamin D status as a result of adipose tissue sequestration [1, 7]. However, the use of vitamin D supplements and multivitamins was less common among overweight and obese children in the present study. Specifically, overweight children were the least likely to use vitamin D supplements and obese children were the least likely to use multivitamins. This association was obtained only in the unadjusted models, indicating that the relationship was confounded by demographic, socioeconomic and lifestyle factors. It is not surprising that we did not see any difference in vitamin D containing supplement use by weight status. The public is likely to be unaware that overweight and obese children need more vitamin D relative to a normal weight child [36]. The additional requirements for those above healthy body weight need to be considered when recommending supplements and more research is needed to establish weight-specific clinical guidelines for vitamin D.

To our knowledge, this is the first population-based study that describes factors associated with the use of vitamin D supplements among children in Canada. Also, only a few studies have examined the determinants of multivitamin supplement use among children. The other strengths of this study were the use of a large provincially representative sample, its high response rate for school-based research, and, the execution of multilevel regression to account for hierarchical data structure and to assist the survey design effect with weighted analysis. There were some limitations of this study. The use of self-reported information was not validated, but, potential bias was minimized by using a validated food frequency questionnaire that has been shown to be comparable in estimating typical intake to multiple 24-hour recalls. Parents did not answer the question about child supplement use and therefore, it is possible that the children may not have known the difference between vitamin D supplements and multivitamins mentioned in the questionnaire in addition to recall bias. However, evaluation assistants helped minimize this issue by providing explanations while children completed the surveys. The present study was conducted using a sample of grade five students in Alberta and the participation rate was 59 %. Therefore, caution is warranted when generalizing results to other children.

## Conclusions

A low proportion of school-aged children were taking vitamin D supplements and most of them did not use them on a daily basis. Physically active children were more likely to use supplements and therefore, parents who encourage their children to be more active may also be more likely to provide them with supplements, or those who were using supplements are more likely to be physically active. Parents with low educational attainment and those who live in rural and urban areas should be the target of campaigns to promote vitamin D supplementation for children. Although overweight and obese children require more vitamin D, vitamin D supplement use was not associated with body weight status. Therefore, nutritionists and health care providers need to consider the weight status of the child when recommending supplements. Therefore, nutritionists and health care providers need to consider the weight status of the child when recommending supplements. Further studies are required to explore other possible determinants of supplement use such as parents’ perceptions on using supplements and family history of supplements use.

## Abbreviations

REAL Kids Alberta: Raising healthy eating and active living kids
YAQ: Harvard youth/adolescent food frequency questionnaire
BMI: Body mass index
PAL: Physical activity level
